# Coiled Encapsulated *Trichinella* Larva

**DOI:** 10.4269/ajtmh.20-0382

**Published:** 2020-11

**Authors:** Julika Kaplan, Christie J. Finch, Jill E. Weatherhead

**Affiliations:** Baylor College of Medicine, Houston, Texas

**Figure 1.**
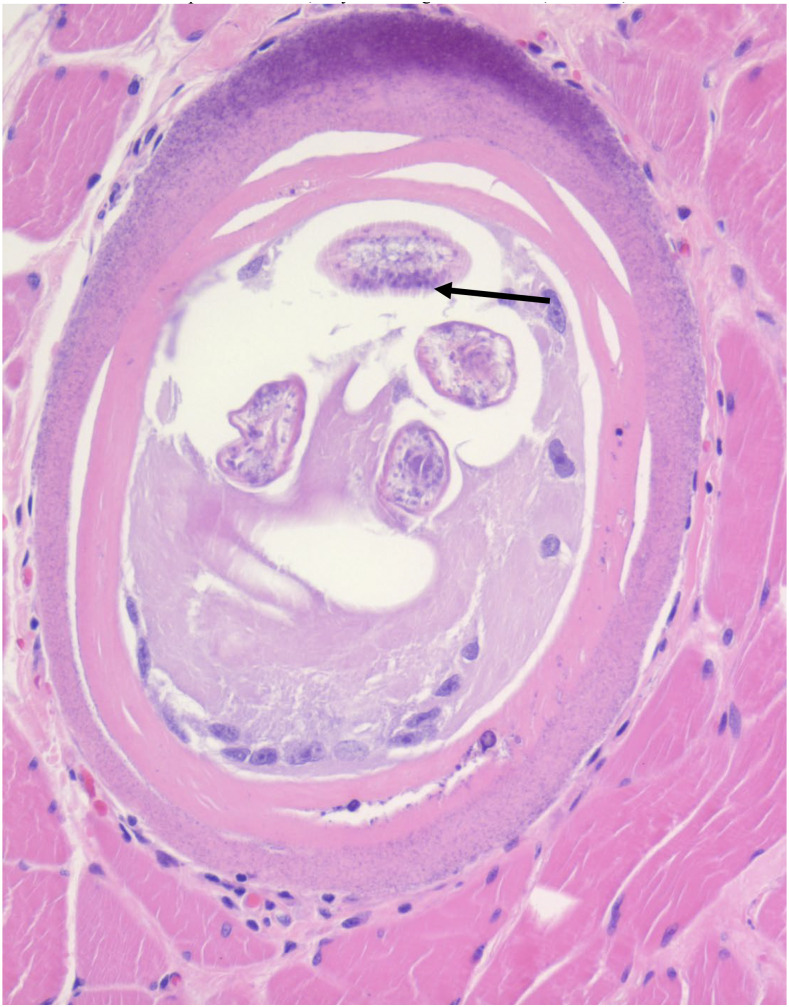
*Trichinella* encapsulated cyst in skeletal muscle. This figure appears in color at www.ajtmh.org.

A 51-year-old man with metastatic papillary thyroid cancer underwent modified neck dissection with excision of the strap muscle. In addition to malignancy, histopathology demonstrated a 275-µm encapsulated cyst containing a larva 27 µm in diameter with bacillary bands (arrow) within the skeletal muscle consistent with *Trichinella* species (Figure 1). Bacillary bands are rows of single gland cells traversing the length of the larva and are described in *Trichinella*, *Trichuris*, and *Capillaria* nematodes.^1^ Several encapsulated cysts with intact larvae, in addition to multiple degenerating lesions of similar size, were seen throughout the muscle near the malignant masses. The patient was originally from Mexico City, Mexico, and moved to Houston, TX, 17 years before presentation without further travel. He ate raw pork 10 years ago while in Texas but denied wild animal consumption. At presentation, white blood cell count was 6,900 cells/µL, absolute eosinophil count was 360 cells/µL, and serum creatine kinase was 149 units/L. Stool ova and parasite examination was negative, serum *Strongyloides* antibody was negative, and serum *Toxocara* antibody was positive. The patient was asymptomatic. Although the patient most likely had chronic trichinellosis, which is less responsive to anthelmintic therapy, he did receive albendazole, and the absolute eosinophil count decreased to 180 cells/µL after treatment. *Trichinella* is transmitted to humans by consumption of raw or undercooked meat of infected animals.^2^ From 2011 to 2015, 80 cases of trichinellosis were reported in the United States; 9–10 occurred in Texas and were related to bear meat consumption.^3^ Trichinellosis from domestic pork is rare in the United States because of extensive legislation and public health campaigns.^2^ Conventionally, trichinellosis is diagnosed based on the presence of classical signs and symptoms including fever, myalgia, edema, and diarrhea after recent consumption of raw or undercooked meat in combination with subsequent positive serologic assay. Serologic assays target IgG to *Trichinella* excretory/secretory product using ELISA, which can be positive 12–60 days after infection. False-negative tests can occur if the infectious load is minimal or if the assay is performed years after initial infection. It should also be noted that serologic assays can yield false-positive results from cross-reactivity with other parasitic infections. Muscle biopsy is less frequently used as a diagnostic tool but has high specificity when positive.^4^
